# Lithium ameliorates neural differentiation restoring cell death balance in Cornelia de Lange syndrome 2D and 3D models

**DOI:** 10.1038/s41420-026-03085-z

**Published:** 2026-03-28

**Authors:** Chiara Parodi, Antonella Lettieri, Paolo Grazioli, Elisabetta Di Fede, Sara Grassi, Esi Taci, Andrea Toscani, Simona Prioni, Stefano Rebellato, Elisa Adele Colombo, Silvia Rasetti, Alessandro Cutarelli, Milena Mariani, Stefania Corti, Palma Finelli, Alessandro Prinetti, Grazia Fazio, Angelo Selicorni, Luciano Conti, Cristina Gervasini, Valentina Massa

**Affiliations:** 1 Department of Health Sciences, Università degli Studi di Milano, Milan, Italy; 2 “Aldo Ravelli” Center for Neurotechnology and Experimental Brain Therapeutics, Università degli Studi di Milano, Milan, Italy; 3 Department of Medical Biotechnology and Translational Medicine, University of Milano, Segrate, Milano, Italy; 4 Tettamanti Center, Fondazione IRCCS San Gerardo dei Tintori, Monza, Italy; 5 Dipartimento di Biologia Cellulare, Computazionale e Integrata - CIBIO, Trento, Italy; 6 U.O. di Pediatria dell’ASST Lariana, Como, Italy; 7 Fondazione IRCCS Ca’ Granda Ospedale Maggiore Policlinico di Milano, Milan, Italy; 8 SS Medical Genetics Laboratory, SC Clinical Pathology, Foundation IRCCS Ca’ Granda Ospedale Maggiore Policlinico, Milan, Italy; 9 School of Medicine and Surgery, University of Milano-Bicocca, 20900 Monza, Italy

**Keywords:** Neurodevelopmental disorders, Translational research

## Abstract

Cornelia de Lange syndrome (CdLS) is a rare genetic disorder that affects almost any organ, including the central nervous system. It leads to a wide range of neurodevelopmental delays, and there are currently no available clinical treatments. CdLS is caused by pathogenic variants in one of the 7 genes coding for the cohesin complex, a multimeric structure responsible for sister chromatid cohesion, or for cohesin ring-interacting proteins. Additionally, altered regulation of molecular pathways during development, including the canonical WNT pathway, can cause CdLS malformations. In our study, we evaluated the positive effects of using lithium as an activator of the canonical WNT pathway to ameliorate neural CdLS phenotype. We have exploited accurate two-dimensional (2D) and three-dimensional (3D) human central nervous system in vitro models representing disease-related neurobiological phenotypes: induced pluripotent stem cells of human origin (hiPSCs) differentiated into neural precursors, neurons, and brain organoids (BOs). CdLS models demonstrate alterations in proliferation and differentiation capabilities when mimicking *HDAC8* haploinsufficiency. Furthermore, RNA-seq analysis of BOs revealed that both neuronal differentiation and the WNT pathway are downregulated when treated with the HDAC8 inhibitor alone. Following lithium treatment, cells show an enhanced ability to differentiate into the neuronal lineage. Additionally, our working hypothesis is that a specific mechanism may exist that, by connecting lipid metabolism, canonical WNT pathway, and cell death, results in typical CdLS neurodevelopmental deficits.

## Introduction

Cell death occurs in numerous tissues during embryonic development, including the central nervous system (CNS). Precisely regulated programmed cell death signaling events play a crucial role during physiological neural development, occurring in a specific spatial and temporal manner to establish the architecture of CNS structures. Abnormalities in this tightly regulated cell process have been observed in the onset of various neurological disorders. However, these forms of cell death can also be triggered pathologically by a range of stimuli and biological events, resulting in uncontrolled differentiation of neuronal cells, thus impacting their functions [1–3].

Indeed, to ensure adequate formation and function of the CNS, the complex development process must be coordinated by the activity of a large number of genes and proteins, including the cohesin complex [4]. Alterations in cognitive, neurological, and behavioral traits of patients with Cornelia de Lange syndrome may be associated with cohesin contribution to morphological abnormalities in hindbrain-derived structures [5], as shown also by MRI analysis of cerebellum malformations [6].

Cornelia de Lange syndrome, CdLS [OMIM #122470, #300590, #610759, #614701, #300882, and #620568], is a rare and complex multisystemic disorder characterized by variable phenotypes. The prevalence of CdLS is estimated to be between 1:10,000 to 1:30,000 live births.

CdLS is characterized by distinctive facial dysmorphism, significant prenatal and postnatal growth retardation, anomalies of the hands and feet, and other associated malformations. Individuals with CdLS present with variable degrees of cognitive impairment attributed to the syndrome’s impact on CNS development. The syndrome evolves with psychomotor delay, difficulties in language acquisition, and autistic-like behavioral issues. CdLS is a genetically heterogeneous disorder, mainly sporadic, caused by dominant autosomal or X-linked de novo pathogenic variants [7]. De novo causative variants have been identified in 6 of the genes involved in chromosome cohesion, specifically, the cohesin complex: nipped-B-like protein (*NIPBL*), structural maintenance of chromosomes 1 A (*SMC1A*) and 3 (*SMC3*), double-strand break repair protein rad21 homolog (*RAD21*), bromodomain-containing protein 4 (*BRD4*), and histone deacetylase 8 (*HDAC8*), but variants in other genes have also been associated with CdLS overlapping phenotypes in CdLS-like patients e.g., ankyrin repeat domain 11 (*ANKRD11*) [8]. Among these genes, *NIPBL*, the most relevant gene in the syndrome, is mutated in about 70% of patients, and *HDAC8* is the second most frequently associated [7].

Currently, no clinical treatment is available for this disorder, and only palliative or surgical treatments are provided for major symptoms.

CdLS patients present abnormal embryonic development, including CNS abnormalities such as microcephaly, cerebellar hypoplasia, and abnormal cortical development [6, 9, 10]. In our previous studies, we pinpointed how cohesins are expressed during CNS development. Our findings revealed that the expression distribution of these proteins increases as the developmental age progresses across all fetal tissues examined, both in mouse and human, with a particularly marked enrichment in neural structures derived from the hindbrain [11]. Interestingly, we also demonstrated a down-regulation of the canonical WNT pathway in *NIPBL*-mutated patient fibroblasts and animal models, suggesting its possible pivotal role in the pathogenesis of CdLS [6, 9, 12, 13].

The WNT signaling pathway plays a key role throughout all stages of CNS development. In addition to orchestrating the organization of the neural plate along the embryonic axis, WNT proteins regulate neural tube formation, which in turn contributes to the development, migration, and maturation of neurons [14–16].

Our and other previous studies have shown that the canonical WNT pathway is indeed perturbed in CdLS models, and this is associated with CNS abnormalities [6, 9, 13, 17].

This study aimed to advance our understanding of the relationship between cohesin dysfunction, alterations in WNT signaling, and central nervous system development in CdLS patients, using both 2D and 3D model systems. Notably, this disrupted molecular network may be at least partially rescued by pharmacological activation of the WNT pathway, for example, through the use of lithium, a known WNT activator.

## Results

### HDAC8 chemical inhibition impairs hNPC differentiation process

**Fig. 1:**
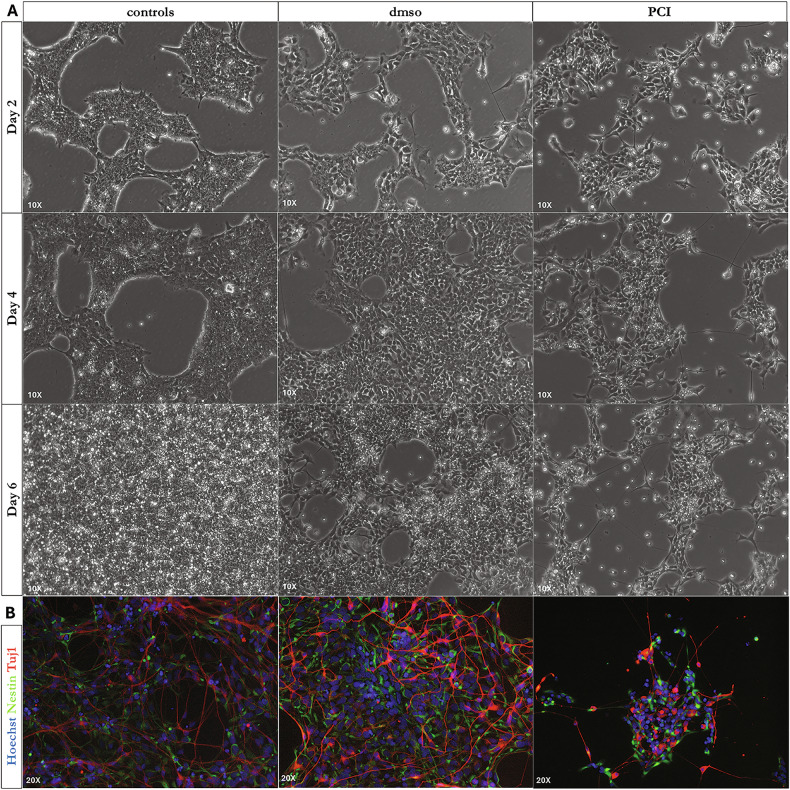
CdLS hNPCs show altered differentiation. **A** hNPCs controls and treatments of proliferating hNPCs with HDAC8 inhibitor (PCI-34051). The panel shows proliferating hNPCs controls and treated with DMSO (as a vehicle for PCI-34051) and PCI-34051 20 μM at 10X magnification. Images were acquired at 3 time points: day 2 (48 h), day 4 (96 h), and day 6 (144 h). **B** Immunofluorescence staining: Hoechst-stained nuclei are shown in blue, Nestin-stained neural precursors in green, and Tuj1-stained mature neurons in red (Supplementary Table 1 and Supplementary Table 2). Images were acquired after 13 days of treatment.

To in vitro mimic CdLS, proliferating hiPSC-derived hNPCs were treated with an HDAC8 inhibitor (PCI-34051, referred to as PCI hereafter) at 20 μM [18–20]. that efficiently mimics the downregulation of HDAC activity on SMC3 (Supplementary Fig. 1). Therefore, we selected the concentration of PCI for our experiments to investigate the effects of HDAC8 deficiency on the maintenance of hNPCs (Fig. 1).

**Fig. 2:**
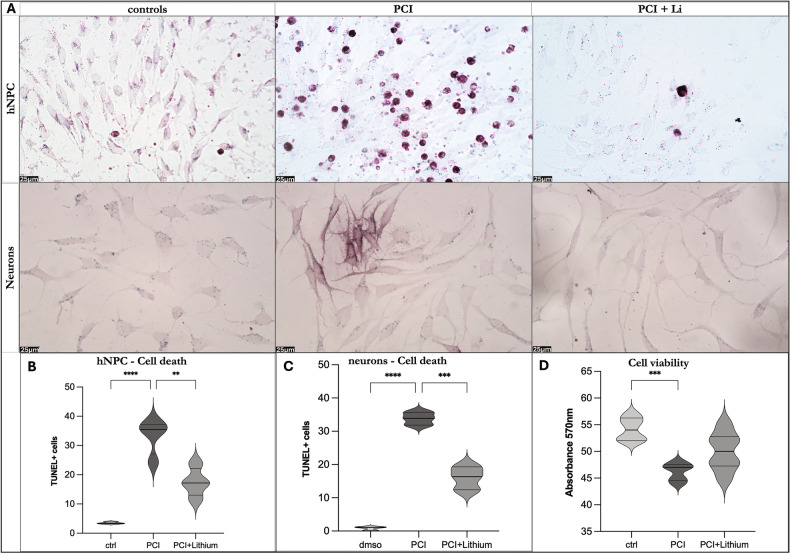
Lithium rescues cell survival in CdLS models by activating WNT signaling. **A** TUNEL assay was used to evaluate the cytotoxicity of HDC8 inhibition in hNPC CdLS models during differentiation compared to controls and co-treatment with lithium. **B** Upon lithium exposure, hNPCs CdLS models show a decrease in cell death compared to cells treated with HDAC8 inhibitor (PCI). One-way ANOVA test Sidak’s correction: PCI vs. ctrl *p* value **** <0,0001; PCI+Lithium vs. PCI *p* value *** 0,0005 (10 images/ condition). **C** Upon lithium exposure, neurons CdLS models show a decrease in cell death compared to cells treated with HDAC8 inhibitor (PCI). One-way ANOVA test Sidak’s correction: PCI vs. ctrl *p* value **** <0,0001; PCI+Lithium vs. PCI p value *** 0,0003 (10 images/ condition). On the axis are reported: the experimental groups (x-axis), and the number of TUNEL-positive cells at 72 h of lithium exposure (y-axis). Images were taken at ×40. **D** MTT assay was used to evaluate the viability of hNPCs during differentiation. Treated cells with the HDAC8 inhibitor (PCI) show significantly reduced viability compared to controls. Upon lithium exposure, cells showed a trend similar to controls. Scale bar represents 25 μm. Bars express Min to Max values. One-way ANOVA test: PCI vs. ctrl ** 0,0050; PCI+Lithium vs. PCI ns 0,2132; PCI+Lithium vs. ctrl ns 0,0504 (6 replicates/condition).

Cells were treated with PCI or DMSO (vehicle control) for six days, and images were acquired at multiple time points during differentiation (Fig. 1A). As shown in Fig. 1A, hNPCs viability appeared to be affected after 48–72 h. However, PCI-treated cells exhibited increased mortality (Fig. 1A and Fig. 2A–D) and reduced confluence compared to both DMSO-treated and untreated controls (Fig. 1A).

To further assess whether HDAC8 inhibition impacts early neuronal differentiation, we analyzed the expression of neural-specific markers in differentiating hNPCs. As shown in Fig. 1B and Supplementary Fig. 2, PCI treatment resulted in reduced Tuj1-positive projections relative to both DMSO and control conditions. Collectively, these findings indicate that HDAC8-deficient hNPCs show increased cell death along with altered proliferation and differentiation, consistent with in vitro phenotypes observed in CdLS models.

### RNA-seq analysis revealed neuronal differentiation and WNT pathway downregulation upon HDAC8 inhibition in brain organoids

**Fig. 3:**
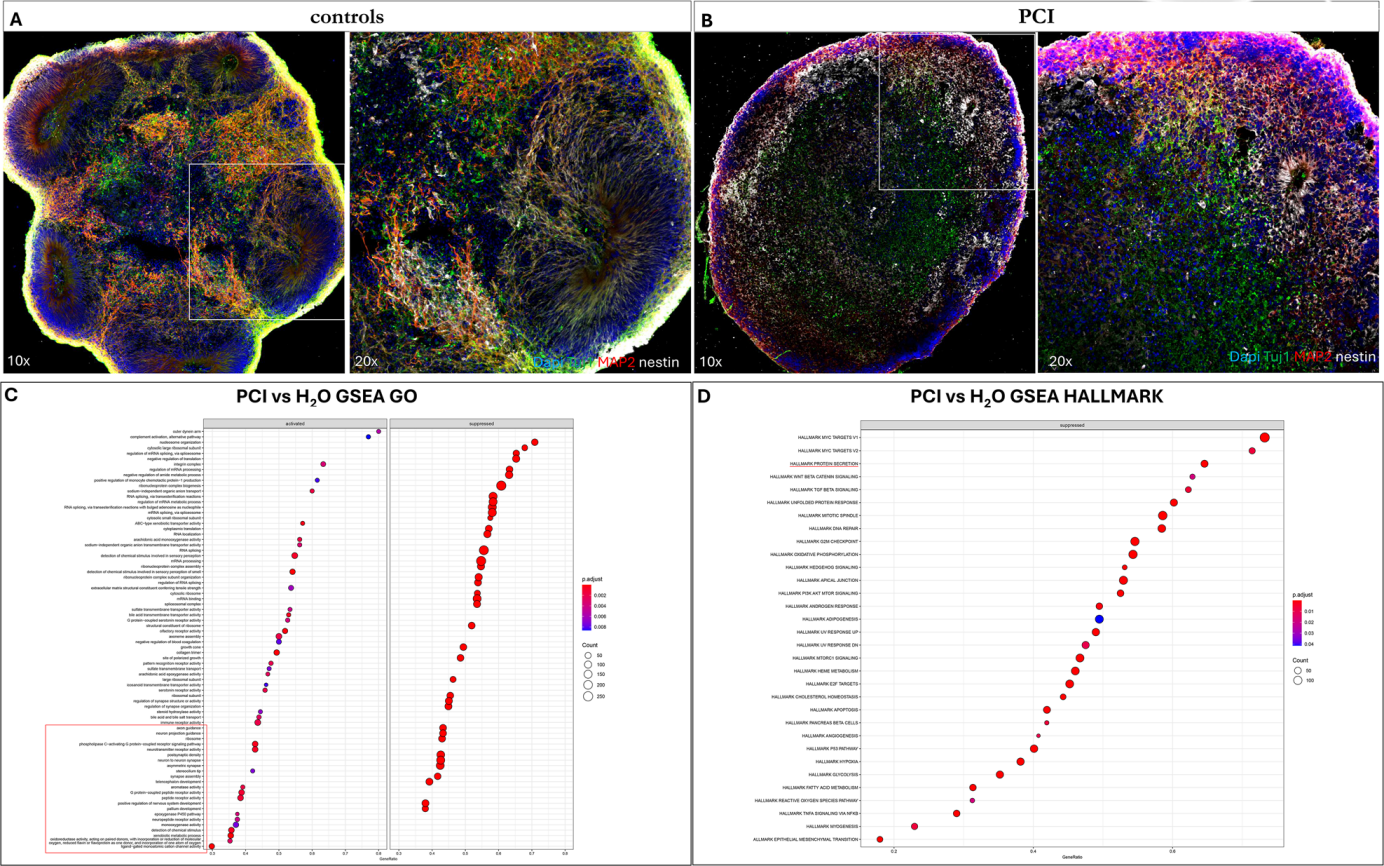
RNA-seq analysis in brain organoids uncovers neuronal differentiation and WNT pathway downregulation. **A**, **B** Characterization of brain organoids at d45 by immunofluorescence experiments; 20 μm brain organoid sections immunolabelled with anti-Nestin antibody (white) and anti-Tuj1antibody (green), and anti-MAP2 (red) at ×10 and ×20 magnification; nuclei were counterstained with DAPI; boxed areas are shown at higher magnification in the corresponding side panel; images were acquired by confocal microscope with 10x and 20x magnification. **A** Control brain organoid; **B** brain organoid treated with PCI-34051 20 μM for 48 h. **C** GSEA GO analysis indicating activated or suppressed pathways involved after HDAC8 inhibition in brain organoids; **D** GSEA HALLMARK analysis shows the suppressed pathways in brain organoids following HDAC8 inhibition.

To further investigate the role of HDAC8 insufficiency in CNS development and neural differentiation, 3D brain organoids (BOs) were generated and treated with PCI (Fig. 3). Compared to controls, BOs subjected to HDAC8 inhibition exhibited markedly impaired differentiation, as demonstrated by disrupted rosette formation and reduced expression of neural markers such as Tuj1 and MAP2 (Fig. 3A, B).

To investigate the downstream effects of HDAC8 inhibition, RNA-seq analysis was performed on control and PCI-treated brain organoids. Performing Gene Set Enrichment Analysis (GSEA), the inhibition of HDAC8 in BOs revealed differentially expressed genes (DEGs) associated with neuronal differentiation (Fig. 3C), which resulted mostly downregulated.

Furthermore, hallmark gene set analysis highlighted consistent expression patterns corresponding to well-defined biological processes, aligning with the observed DEGs. Notably, treated organoids showed significant enrichment of downregulated genes within the WNT signaling pathway, indicating that HDAC8 inhibition results in suppression of WNT activity compared to controls.

In our work, BOs models demonstrate the downregulation of genes associated with the WNT pathway following HDAC8 inhibition (Fig. 3D).

### Lithium ameliorates the CdLS hNPC and BO phenotype during neuronal differentiation

**Fig. 4:**
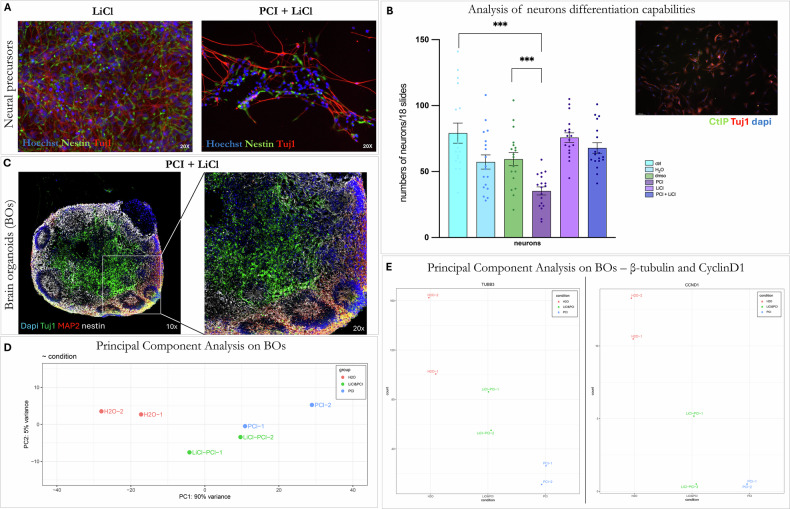
Lithium restores differentiation capabilities in the CdLS model. **A** Differentiating hNPCs into neuronal-like cells treated with LiCl 3 mM or LiCl 3 mM + PCI- 34051 20 μM (compared with controls and PCI shown in Fig. 1) at ×20 magnification. Images were acquired after 13 days of treatment. Hoechst-stained nuclei are shown in blue, Nestin-stained neural precursors in green, and Tuj1-stained mature neurons in red. **B** Analysis of the effects of the HDAC8 inhibitor PCI34051 and lithium exposure on the differentiation capabilities of the hiPSCs into neurons (representative image of neurons shown on the side of the graphs). Inhibition of HDAC8 significantly impacts the neurons’ differentiation capabilities compared to controls. Light blue: neurons control; blue: neurons treated with H20 (lithium vehicle); green: neurons treated with DMSO; purple: neurons treated with PCI34051; light purple: neurons treated with LiCl; dark blue: neurons treated with PCI34051 and LiCl. Student *t* test *p* < 0.1234 (ns), *p* < 0.0332 (*), *p* < 0.0021 (**), *p* < 0.0002 (***), *p* < 0.0001 (****) and values are expressed as means ± SEM. **C** Characterization of brain organoids treated with PCI34051 and/or LiCl at d45 by immunofluorescence experiments (compared with controls and PCI shown in Fig. 2); 20 μm brain organoid sections were immunolabelled with anti-Nestin antibody (white), anti-Tuj1antibody (green), and anti-MAP2 (red); nuclei were counterstained with DAPI; boxes indicate areas shown at higher magnification to the corresponding side panel; images were acquired by confocal microscope with ×10 and ×20 magnification. **D** Principal Component Analysis (PCA) plots of transcriptomic analysis of BOs control and treated with PCI34051, alone or combined with LiCl (PCI34051+LiCl), showing the population variance comparison between PCI34051 with control and PCI34051-treated BOs; **E** differential *TUBB3* and *CCND1* expression in BOs.

During the differentiation process of hNPCs into neurons, cells were treated with LiCl and/or an HDAC8 inhibitor (for modeling CdLS) for 13 days. Immunostaining for Nestin and Tuj1, markers for neural precursors and immature neurons, respectively, revealed that LiCl treatment increased the rate of neuronal differentiation, resulting in a higher number of Tuj1-positive cells, compared to the observed CdLS phenotype (Fig. 1). Nonetheless, in the presence of LiCl (Fig. 4), the morphology of Tuj1-positive cells in the double treatment seems partially restored (Fig. 4A). This suggests that the LiCl treatment could rescue the hNPC phenotype during neuronal differentiation impaired upon HDAC8 inhibition.

Interestingly, co-treatment with LiCl and the tested HDAC8 inhibitor partially restored the differentiation capability of the cells, despite the high mortality rate caused by the presence of the inhibitor (Figs. 1B, 2A–D, and 4A).

Then, to see if HDAC8 inhibition could also affect later stages of neural differentiation, hiPSCs were differentiated into neurons and treated with PCI. HDAC8 inhibition significantly affects the neurons’ ability to differentiate. Once again, co-treatment with LiCl restored neuron differentiation capability (Fig. 4B).

Moreover, in Fig. 4B we show that upon LiCl exposure, our 3D model (BOs) displayed an ameliorated phenotype (Fig. 4C), exhibiting a capability for neuronal differentiation similar to the controls (Fig. 3A).

We applied dimensionality reduction using principal component analysis to generate signatures (principal components) that separate the trajectories of BOs controls, BOs treated with the HDAC8 inhibitor, and BOs treated with the HDAC8 inhibitor plus lithium.

Principal Component Analysis (PCA) on transcriptomic data indicates that the sample group treated with the HDAC8 inhibitor responds differently compared to the control group (Fig. 4D, E). Notably, after treatment with lithium, a recovery is observed that resembles the control group. This finding is also supported by our evaluation of the β-tubuline III (TUBB3) pathways and the analysis of CyclinD1 (CCND1) (Fig. 4D, E).

### GM3 sphingolipid increases upon HDAC8 inhibitor treatment in neurons

Another potential player with a crucial role in cell death regulation is sphingolipids, which are present in cell membranes. Sphingolipids directly support essential biological functions, including cell motility, growth, senescence, and differentiation, as well as determining cell fate, such as survival or death. Particularly, ceramide is known to induce apoptosis [21]. In addition, gangliosides, a class of sphingolipids abundant in the CNS, have been implicated in a wide range of biological functions, including the regulation of cell proliferation and death. Among them, studies suggest that monosialodihexosylganglioside (GM3) has a role as a modulator of in vivo neuronal cell death [22].

In order to investigate the involvement of sphingolipid metabolism in neuronal processes, we studied it in relation to neuronal differentiation in our in vitro model of CdLS.

**Fig. 5:**
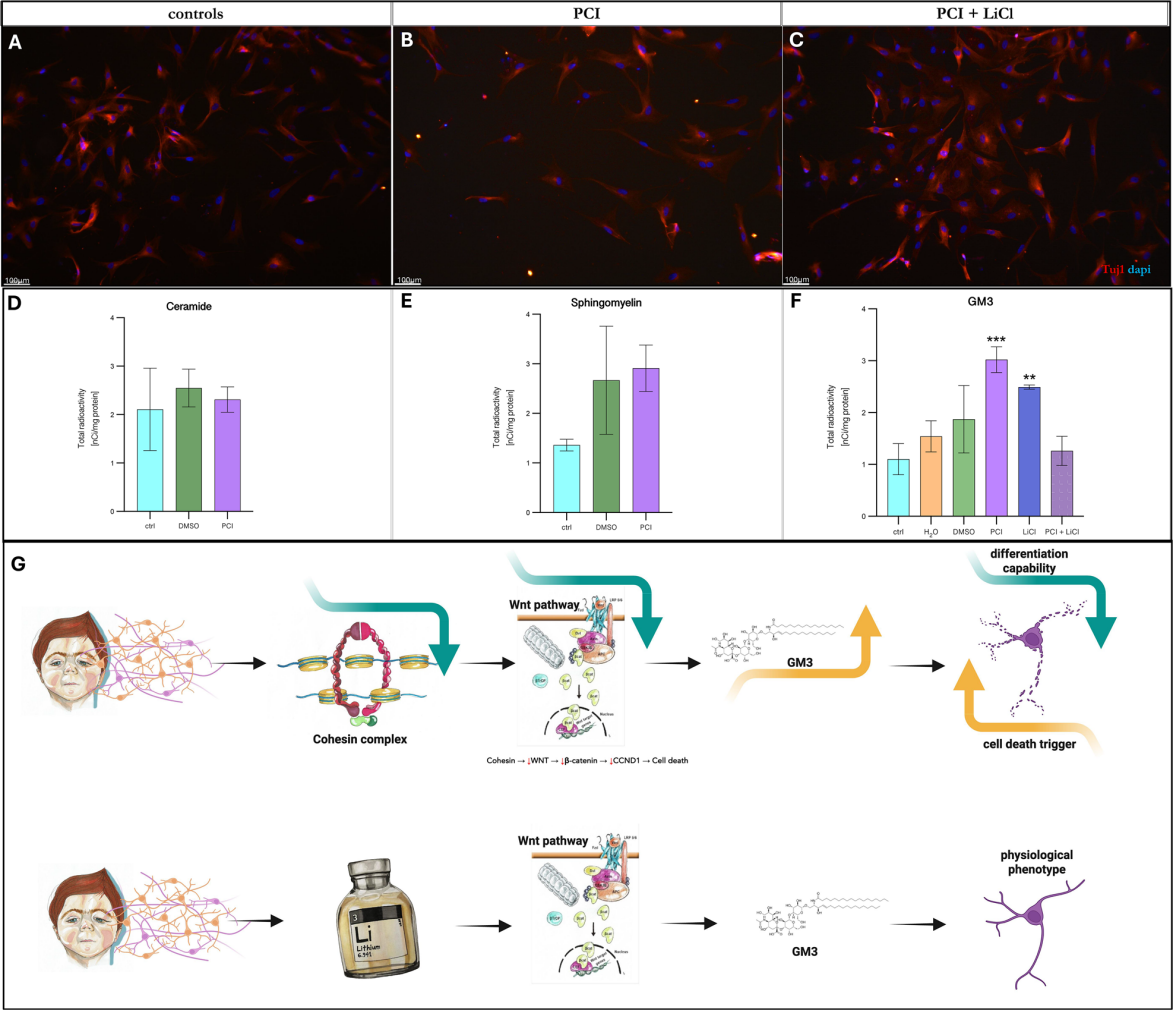
GM3 sphingolipid levels in CdLS neuronal model. Immunofluorescence staining showing differentiated neurons control from iPSC (**A**), differentiated neurons treated with the HDAC8 inhibitor PCI-34051 20 μM (**B**), and differentiated neurons treated with LiCl 3 mM + PCI-34051 20 μM (**C**) at ×20 magnification. Scale bar 100 μm; DAPI-stained nuclei are shown in blue and Tuj1-stained mature neurons in red. Sphingolipid analysis showing ceramide (**D**), sphingomyelin (**E**) and GM3 (**F**) levels in iPSC-derived neurons treated with PCI-34051 20 μM; control in light blue; neurons treated with H20 (LiCl vehicle) in yellow; treated with DMSO in green; neurons treated with LiCl in blu; neurons treated with PCI34051 in light purple; neurons treated with PCI34051 and LiCl in purple. **D** Ceramide analysis shows no difference with PCI34051 treatment; **E** sphingomyelin analysis shows no difference with PCI34051 treatment; **F** GM3 analysis shows a significant increase in GM3 levels with PCI34051 treatment (** *p* < 0.005 vs control) and restored levels comparable to control upon LiCl treatment; Student *t* test *p* < 0.1234 (ns), *p* < 0.0332 (*), *p* < 0.0021 (**), *p* < 0.0002 (***), *p* < 0.0001 (****); and values are expressed as means ± SD (**G**) graphical representation of working model.

We first confirmed in our model the statistically significant impairment in neural differentiation upon PCI treatment and the partial rescue of Tuj1-positive cells when cotreated with LiCl (Fig. 5A–C).

Then, we performed analyses of the abundance of sphingolipid in neurons derived from hiPSCs and treated with the HDAC8 inhibitor, as well as with lithium.

Our data indicate that no difference was observed in ceramide or sphingomyelin levels (Fig. 5D, E). Intriguingly, a significant increase in GM3 levels subsequent to treatment with the HDAC8 inhibitor was observed. In the samples treated with PCI, a 173.8% increase in GM3 level was observed compared to the control group. In the samples treated with lithium, there was a 126.2% increase compared to the control group (Fig. 5F). Hence, a connection among cell death, WNT pathway, lipid metabolism could explain neurodevelopmental abnormalities in CdLS, restored in 2D and 3D models by lithium (Fig. 5G, created with Biorender).

## Discussion

This study provides further evidence for the involvement of the canonical WNT pathway in the pathogenesis of CdLS. We employed an in vitro hiPSC-based model, a versatile platform for investigating neural development through directed lineage differentiation. Building on our previous work, we demonstrated that disruption of the cohesin complex can be recapitulated in this human cellular system by pharmacologically inhibiting HDAC8 deacetylase activity using a selective chemical compound [17]. HDAC8 is a histone deacetylase that influences SMC3 availability, making it a key player in the functioning of the cohesin complex. An association has been found between a chemical inhibition of HDAC8, which resulted in the establishment of CdLS model, and increased cell death in developing neural tissues, both in vitro and in vivo, with a consequent reduction in neuronal differentiation capabilities [17]. This may contribute to the severe mental disability seen in CdLS patients. In parallel, we chemically activated the canonical WNT pathway. Subsequent analyses revealed that WNT pathway activation influenced the hNPC phenotype, enhancing the differentiation capacity of cells in which HDAC8 activity had been inhibited by chemical treatment. Using the same cellular model, we treated hiPSC-derived hNPCs during neuronal differentiation to evaluate their ability to successfully mature into neurons and to determine the impact of HDAC8 inhibition on this process. As anticipated, HDAC8 inhibition disrupted proper neuronal differentiation. Notably, this impairment was partially rescued by activating the canonical WNT pathway through LiCl treatment. Consistent with these findings, our team previously observed similar rescue effects in murine neural stem cells and patient-derived lymphoblastoid cell lines [6, 17]. Notably, the LiCl treatment outcome remains consistent in human iPSC-derived cells.

Brain organoids have recently emerged as a promising tool for studying neurodevelopment and brain diseases in vitro [23–25]. This model is considered biologically relevant as it recreates, to some extent, the complex architecture and diverse cell type composition found in vivo, enabling a more informative analysis of biological processes related to illness and therapeutic interventions.

Here, we investigated the BOs transcriptome after PCI and LiCl treatment, with the dual objectives of modeling CdLS and exploring a new therapeutic strategy. After confirming the effects of HDAC8 inhibition at the transcriptional level, we assessed the impact of LiCl combined with PCI compared to inhibitor exposure alone or with the control. Upon PCI treatment, we identified a clear difference supported by a consistent number of DEGs. Interestingly, among the deregulated pathways, we found many genes associated with neural specification patterning (Fig. 3C), and further WNT/-catenin signal results suppressed (Fig. 3D). Notably, PCA results showed that the combined treatment with PCI and LiCl clustered with the control rather than with the samples exposed to PCI alone, suggesting that WNT pathway activation could reverse the effects caused by HDAC8 inhibition. It will be important to strengthen these findings to assess effects with complementary approaches such as other chemical or genetic models.

Additionally, in vitro cultured neurons are a valuable tool for drug studies, as they lack a fully formed blood-brain barrier, which allows researchers to observe the effects of drugs on neural tissue more easily [26].

Neuronal death, predominantly via apoptosis, is a common feature in many neurodegenerative diseases. Studies suggest that elevated ceramide levels play a significant role in mediating these processes [21].

It has been established for over 30 years that the mechanism of action of certain antitumor drugs, which induce cell death at least in part via apoptosis, involves the stimulation of increased ceramide levels [27]. In the CNS, the principal source of ceramide is the hydrolysis of a more complex sphingolipid, known as sphingomyelin. In cases of severe depression, which falls under major mood disorders, there is an increase in ceramide levels linked to heightened activity of the acid sphingomyelinase enzyme. The rise in ceramide levels has significant implications, as it not only contributes to increased cell death but also affects various other processes, including a reduction in adult neurogenesis that, in turn, is believed to play a crucial role in neurodepressive disorders [28].

When considering the relationship between sphingolipids and apoptosis, ceramide is often the primary focus. However, in the present study we observed an important pattern for GM3. Indeed, other studies have also shed light on the roles of GM3 in the context of apoptosis [22].

While GM3 is generally less represented in the central nervous system, its significance as a ganglioside should not be underestimated. Current research indicates that no two neurons exhibit identical lipid compositions or the same functional roles for these lipids [29].

Importantly, the literature suggests a role for GM3 in the inhibition of Wnt/β-catenin signaling [30]. Whether GM3 can be considered as a possible new player and highlight its link with CdLS etiopathogenesis warrants further studies in the future, including exploring the mechanism in other cohesin models and in genetically manipulated systems, for better representing human patients and specific pathogenetic variants.

This work further highlights the pivotal role of cohesin genes in the central nervous system, acting through the canonical WNT pathway. We show that cohesin complex disruption affects normal neuronal differentiation from early stages, impacting cell death regulation.

## Materials and methods

### Human-induced pluripotent stem cells (hiPSCs)

This study utilized commercially available hiPSCs, specifically the “Human Episomal iPSC Line” (Gibco #A18945), and CS5NXHiCTR-nxx line (kindly provided by Prof. Stefania Corti), for in vitro experiments. More details in the Supplementary materials.

### Human neural precursor cells (hNPC)

We used a commercially available kit called “PSC Neural Induction Medium” (Gibco #A1647801) to generate hNPCs. We started with high-quality hiPSCs, which were 70–80% confluency and had minimal or no differentiated colonies (more detailed in Supplementary).

### Differentiation into the neuronal lineage

hNPCs were treated with a homemade Neuronal Differentiation Medium specifically designed to induce differentiation through the neuronal lineage [31, 32]. Details are reported in the Supplementary Materials.

### Cells treatments

The cells underwent treatment with two different chemical molecules: LiCl (a canonical WNT pathway activator) and PCI-34051 (an HDAC8 inhibitor) (more details in Supplementary).

### Immunofluorescence

Immunofluorescent stainings on cells and BOs were performed as described in the Supplementary Materials.

### Steady state labeling with [1-^3^H]sphingosine and lipid analysis

[1-^3^H]sphingosine (radiochemical purity over 98%; specific radioactivity of 1.36 Ci/mmol) was prepared by specific chemical oxidation of the primary hydroxyl group of sphingosines followed by reduction with sodium boro[^3^H]hydride as previously described [33].

Cell sphingolipids were steady-state metabolically labeled with 3×10-8 M [1-^3^H]sphingosine (2 h pulse/48 h chase) as described previously [34]. After 48 hours, cells were collected, centrifuged and lysed in ice-cold water. Following lyophilization, lipids were extracted with chloroform/methanol/water (2:1:0.1, by volume), subjected to two-phase partitioning, and radioactive lipids were separated by monodimensional

High-Performance Thin-Layer Chromatography (HPTLC) and quantitatively analyzed by digital autoradiography [35].

### Brain organoids (BOs)

Brain organoids were derived from an hiPSC line kindly provided by Prof. Corti’s laboratory, using a modified version of the published protocols, as previously described [36]. On day 45, the BOs were subjected to treatments and then collected (Supplementary materials).

### RNA sequencing

BOs derived from hiPSC controls have been used in RNA-seq experiments following exposure to PCI-34051, with analyses conducted both with and without lithium exposure and control.

BO pellets have been snap-frozen, and RNA was extracted with Trizol (Sigma Aldrich, Italy) following the manufacturer’s protocol. Sequencing was performed as described in Supplementary Materials.

### MTT assay

The MTT tetrazolium reduction assay for cell viability was conducted on hNPC cells during their differentiation, following treatments with PCI and PCI combined with lithium (Supplementary materials).

### TUNEL assay

Apoptosis rate in hNPCs and neurons was evaluated using Terminal deoxynucleotidyl transferase (TdT) dUTP Nick-End 364 Labeling (TUNEL) assay (Supplementary materials).

### Protein extraction and western blot analysis

To assess the effect of acetylation on the cohesion target, hNPCs protein extraction and Western blotting using an anti-acetylated SMC3 antibody were performed as previously described [36–38]. and detailed in the Supplementary materials.

### Statistical analyses

Data was analyzed using Prism software (GraphPad Software v.10.2.2) and expressed as mean ± Standard Error of the Mean (SEM) or ± Standard Deviation (SD). Appropriate post-analysis corrections were applied when needed (details in legends). Statistical analysis was performed using two-tailed *Student* *t* test or One-way ANOVA test where appropriate, considering significance for *p* value < 0.05 (* *p* < 0.05; ** *p* < 0.01; *** *p* < 0.001).

## Supplementary information

Supplementary Material

whole membrane WB

## Data Availability

The datasets generated during and/or analysed during the current study, in particular the raw FASTQ sequences, are available in the BioStudies-ArrayExpress database (https://www.ebi.ac.uk/biostudies/arrayexpress) under accession number E-MTAB-15615.
