# Supplementary material for: Lithium ameliorates neural differentiation restoring cell death balance in Cornelia de Lange syndrome 2D and 3D models
Source: Cell Death Discov. 2026 Mar 28;12:203. doi: 10.1038/s41420-026-03085-z (PMC13150035; doi:10.1038/s41420-026-03085-z)
Supplement: Supplementary file 1 — Supplementary Material [file 41420_2026_3085_MOESM1_ESM.docx]

**Supplementary materials**

**Materials and methods**

***Human-induced Pluripotent Stem Cells (hiPSCs*)**

This study utilized commercially available hiPSCs, specifically the "Human Episomal iPSC Line" (Gibco #A18945), for *in vitro* experiments. These cells were derived from CD34^+^ cord blood using a three-plasmid, seven-factor (SOKMNLT; SOX2, OCT4 (POU5F1), KLF4, MYC, NANOG, LIN28, and SV40L T antigen) EBNA-based episomal system. The hiPSC line is considered to have a zero footprint, as it was not integrated into the genome during the reprogramming process. The cells also exhibit a normal karyotype and endogenous expression of pluripotent markers such as Oct4, Sox2, Nanog, SSEA4, TRA-1-60 and TRA-1-81.

During the experiments, the hiPSCs were cultured on Geltrex-coated (Gibco #A1413301) 6 multi-well plates using Essential 8 medium (Gibco #A1517001). The medium was replaced daily after the cells were thawed. Passaging and dissociation were performed using Ultrapure EDTA diluted at 0.5 mM (Invitrogen #15575020) in DPBS without Ca^++^ and Mg^++^ (Gibco #14190144). The hiPSCs were maintained at 37°C in a humidified incubator with 5% CO2.

Another commercially available line, CS5NXHiCTR-nxx, has been used. This is a healthy donor hiPSC line that was kindly provided by Prof. Stefania Corti. Both cell lines have been tested for mycoplasma contamination, yielding negative results.

***Human Neural Precursor cells (hNPC*)**

We used a commercially available kit called "PSC Neural Induction Medium" (Gibco #A1647801) to generate hNPCs. We started with high-quality hiPSCs, which were 70-80% confluency and had minimal or no differentiated colonies. As described above, we passed these cells to reach a seeding density of 3x10^5^ hiPSCs/well. After 24 hours, we changed the medium from Essential 8 to PSC Neural Induction Medium and continued to change it every 2 days. After one week, the cells were nearly 100% confluent and ready for harvesting and expansion.

***Differentiation through the neuronal lineage***

Human neural precursor cells (hNPCs) were treated with a homemade Neuronal Differentiation Medium specifically designed to induce differentiation through the neuronal lineage. This medium contains Neurobasal Medium (Gibco #21103049), GlutaMAX Supplement 1X (Gibco #35050061), MEM Non-Essential Amino Acids 1X (Gibco #11140035), B-27 Supplement 1X (Gibco #17504044), BDNF 20 ng/mL, GDNF 20 ng/mL and L-ascorbic acid 200 μM. The cells were then plated on laminin-coated plastic (10 μg/mL), and the medium was changed once every 2 to 3 days.

***Cells treatments***

The cells underwent treatment with two different chemical molecules alone and in combination: LiCl (a canonical WNT pathway activator) and PCI-34051 (an HDAC8 inhibitor). LiCl was dissolved in water (used as a vehicle) with a final concentration of 3 mM. PCI-34051 was dissolved in DMSO (used as a vehicle) with a final concentration of 10 μM and 20 μM. The treatments were freshly added every 48 to 72 hours, and the medium was changed accordingly. Treatments of differentiated neurons from iPSC with LiCl and/or HDAC8 inhibitor (PCI-34051), seeding 10.000 cells/well in 24well at day0 of neuron differentiation protocol.

***Immunofluorescence***

Cells and BOs were fixed with 4% paraformaldehyde (PFA) for 15 minutes at RT or 1 hour and 30 minutes at 4°C, respectively. Then, BOs were cryopreserved in 30% sucrose overnight; the following day, they were embedded in OCT and stored at –80°C until use. Sections of 20 μm were obtained by cryostat. Immunofluorescent stainings were performed to verify the correct generation of hNPCs from hiPSCs and to assess the differences in neuronal maturation. Briefly, after PFA, three washes with Phosphate-Buffered Saline (PBS), cells were permeabilized with PBS Triton X-100 (PBT) 0,5 % for 15 minutes, and then aspecific sites were blocked with PBT 0,3 % + Fetal Bovine Serum (FBS) 2 %, for 1 hour at RT. Incubation with the primary antibody (Supplementary Table 1) was performed overnight at 4°C. The following day, cells were washed and incubated with secondary antibodies (Supplementary Table 2) for 2 hours.

BOs incubation with primary antibodies TUJ1, MAP2 and Nestin was performed overnight at 4°C. Both BOs and neurons were then washed and incubated with secondary antibodies for 2 hours.

Nuclei staining was performed with Hoechst 33342 (hNPCs) and DAPI (BOs). Cells were visualized in PBS with 10 mM sodium azide. BO’s images were acquired at a confocal microscope at the UNIMI facility.

***Steady state labeling with [1-^3^H]sphingosine and lipid analysis***

[1-^3^H]sphingosine (radiochemical purity over 98%; specific radioactivity of 1.36 Ci/mmol) was prepared by specific chemical oxidation of the primary hydroxyl group of sphingosines followed by reduction with sodium boro[^3^H]hydride as previously described [1].

Cell sphingolipids were steady-state metabolically labeled with 3x10-8 M [1-^3^H]sphingosine (2 h pulse/48 h chase) as described previously [2]. After 48 hours, cells were collected, centrifuged and lysed in ice-cold water. Following lyophilization, lipids were extracted with chloroform/methanol/water (2:1:0.1, by volume), subjected to two-phase partitioning, and radioactive lipids were separated by monodimensional HPTLC and quantitatively analyzed by digital autoradiography[3]. Experiments were performed in technical triplicate.

***Brain Organoids (BO****)*

Brain organoids were derived from an iPSC line kindly provided by the Prof. Corti Lab, using a modified version of the Lancaster protocol, as previously described[4]. In brief, 10,000 cells per well were seeded in a U-bottom 96 low-attachment multiwell using E8 medium supplemented with a ROCK inhibitor (10 μM) and centrifuged for 5 minutes at 270 g (day 0). On day 2, the culture medium was replaced with E8 medium containing SB-431542 (10 μM) and Dorsomorphin (5 μM). Subsequently, the medium was completely replaced every two days until day 6. From day 6 to day 22, BOs were cultured in Neurobasal medium supplemented with Glutamax (1X), penicillin-streptomycin (P/S) (1%), and B27 supplement (devoid of Vitamin A) (50X) and were further supplemented with epidermal growth factor (EGF) (20ng/mL) and fibroblast growth factor-2 (FGF-2) (20ng/mL). On day 12, the BOs were transferred to a 24-well low-attachment plate, with half of the medium changed every 2 to 3 days. Subsequently, from day 22 to day 45, the COs were cultured in Neurobasal medium with Glutamax (1X), P/S (1%), B27 supplement (devoid of Vitamin A) (50X), and were supplemented with brain-derived neurotrophic factor (BDNF) (20ng/mL), neurotrophin-3 (NT-3) (20ng/mL), ascorbic acid (AA) (200uM), Dibutyryl cAMP sodium salt (D-cAMP) (50uM), and Docosahexaenoic Acid (DHA) (10uM). From day 45 onward, the BOs were maintained in a Neurobasal medium supplemented with B27 (50X). On day 45, the BOs were subjected to treatments and then collected.

***RNA sequencing***

BO pellets have been snap-frozen, and RNA was extracted with Trizol (Sigma Aldrich, Italy) following the manufacturer’s protocol.

BOs derived from hiPSC controls have been used in RNA-seq experiments following exposure to PCI-34051, with analyses conducted both with and without lithium exposure. The experiments utilized the Universal RNA-Seq kit (Tecan) with targeted transcript depletion using AnyDeplete (Tecan) for ribosomal and globin genes. The final libraries' yields were assessed using a Qubit 4.0 fluorimeter, and their sizes were determined using an Agilent Bioanalyzer. Paired-end sequencing on the NextSeq2000 Illumina platform, 2 × 50, was used to analyze the libraries. Sequencing was performed at 40M reads per sample. The raw FASTQ sequences are available in the BioStudies-ArrayExpress database (https://www.ebi.ac.uk/biostudies/arrayexpress) under accession number E-MTAB-15615. The quality of the raw FASTQ sequences was assessed using FastQC tool (Babraham Institute), and the sequences were aligned against the GRCh38/hg38 reference human genome using the splice-aware aligner STAR v2.7.10b [5]. Sorted, indexed BAM alignment files were utilized for quantification with featureCounts (v2.0.0)[6], considering only uniquely mapped reads. The GRCh38 Ensembl Release 108 annotation was used as a reference. The Bioconductor package DESeq2 v1.30 [7] was used to perform differential gene expression analysis, utilizing custom shell and R scripts. Significant gene sets were selected based on a nominal p-value of less than 0.05 and an absolute log2 fold change greater than 0.58, which translates to an actual fold change higher than 1.5 or lower than 0.67.

Following the identification of differentially expressed genes among all the different genotyped and treated neurons, molecular mechanisms were investigated through gene set enrichment analysis (GSEA) to identify differentially enriched signaling pathways.

***MTT assay***

The MTT(3-(4,5-dimethylthiazol-2-yl)-2,5-diphenyltetrazolium bromide) tetrazolium reduction assay for cell viability was conducted on hNPC cells during their differentiation, following treatments with PCI and PCI combined with lithium. The MTT substrate, prepared in a physiologically balanced solution, was added to the cultured cells at a final concentration of 0.5 mg/ml. The cells were then incubated for 2 hours. The quantity of formazan produced, which is presumably directly proportional to the number of viable cells, was measured by recording changes in absorbance at 570 nm using a plate reader spectrophotometer.

***TUNEL assay***

Apoptosis rate in hNPCs and neurons was evaluated using Terminal deoxynucleotidyl transferase (TdT) dUTP Nick-End 364 Labeling (TUNEL) assay, designed to detect apoptotic cells during the late stages of apoptosis, as previously described[8]. Briefly, slices were fixed in 4% PFA for 10 minutes at RT, then washed three times in PBS for 5 minutes each. Staining for apoptotic cells was performed using the AP-In Situ Cell Death Detection Kit (Roche Diagnostics, Penzberg, Germany) following the manufacturer’s protocol. Slides were mounted for microscopic imaging with homemade glycerol based mounting media with anti fading (DABCO). Apoptotic cells were detected with NanoZoomer-XR Digital slide scanner (Hamamatsu, Japan) for blinded counting.

**Protein extraction and western blot**

hNPCs were washed in PBS, the dry samples were resuspended in cold S300 buffer (50 mM HEPES pH 7.6, 300 mM NaCl, 0.1% NP40, 2 mM MgCl2, 10% glycerol) supplemented with a protease inhibitor cocktail (#P8340, Sigma-Aldrich) and benzonase nuclease (#SC-202391, Santa Cruz Biotechnology), and left on ice for 1h. Samples were centrifuged at maximum speed (13,000 rpm) for 10 min at 4°C, and the supernatant was quantified with Bradford assay following the manufacturer’s instructions. Protein samples were denatured in Laemmli sample buffer 4x (LSB, #1610747, Bio-Rad) supplemented with β-mercaptoethanol (#1610710, Bio-Rad) and boiled for 10 min at 100°C prior to gel loading.

Protein samples were separated by SDS-PAGE (Running buffer 1x diluted from 10x made of 3% Tris HCl, 14,4% Glycine and 1% SDS) and transferred to nitrocellulose membranes (#10600003, Cytiva) in Transfer buffer 1x (20% methanol and 10% Transfer buffer 10x, composed of 3% Tris HCl and 14,4% Glycine). Membranes were washed with TRIS-buffered saline (TBS 1x diluted from 10x made of 3% Tris HCl, 8,7% NaCl and 0,2% KCl) supplemented with 0.1% Tween (TBS-T), blocked for 1h at RT with 5% milk in TBS-T and then with primary antibody diluted in blocking solution or TBS-T for 1h at RT (mouse anti-βactin, 1:1000, SIGMA #A2228; anti-H3, 1:, Abcam #ab1791) or overnight at 4°C (rabbit anti-acetylated SMC3, 1:500, MLB #pd040). Membranes were washed in TBS-T and incubated with goat anti-rabbit or anti-mouse IgG horseradish peroxidase (HRP)-conjugated antibodies (#1706515 and #1706516, Bio-Rad) diluted in blocking solution or TBS-T for 1h at RT. After membrane washes, chemiluminescence signals were detected through ECL incubation (#1705061, Bio-Rad and #RPN2232, Cytiva) and captured by a Chemidoc Imaging System. Data obtained from western blots were analyzed by Image Lab Software and expressed as the ratio between values from proteins of interest and from housekeeping markers (βactin or H3).

**Supplementary Tables**

| Target | Host | Dilution |
| --- | --- | --- |
| Nestin | Mouse | 1:300 |
| Tuj1 | Rabbit | 1:500 |

**Supplementary** Table 1. Primary antibodies for immunofluorescence staining on hNPCs.

| Target | Conjugation | Host | Dilution |
| --- | --- | --- | --- |
| Mouse | 488 | Goat | 1:500 |
| Rabbit | 568 | Donkey | 1:500 |

**Supplementary** Table 2. Secondary antibodies for immunofluorescence staining on hNPCs.

*Supplementary Figures*


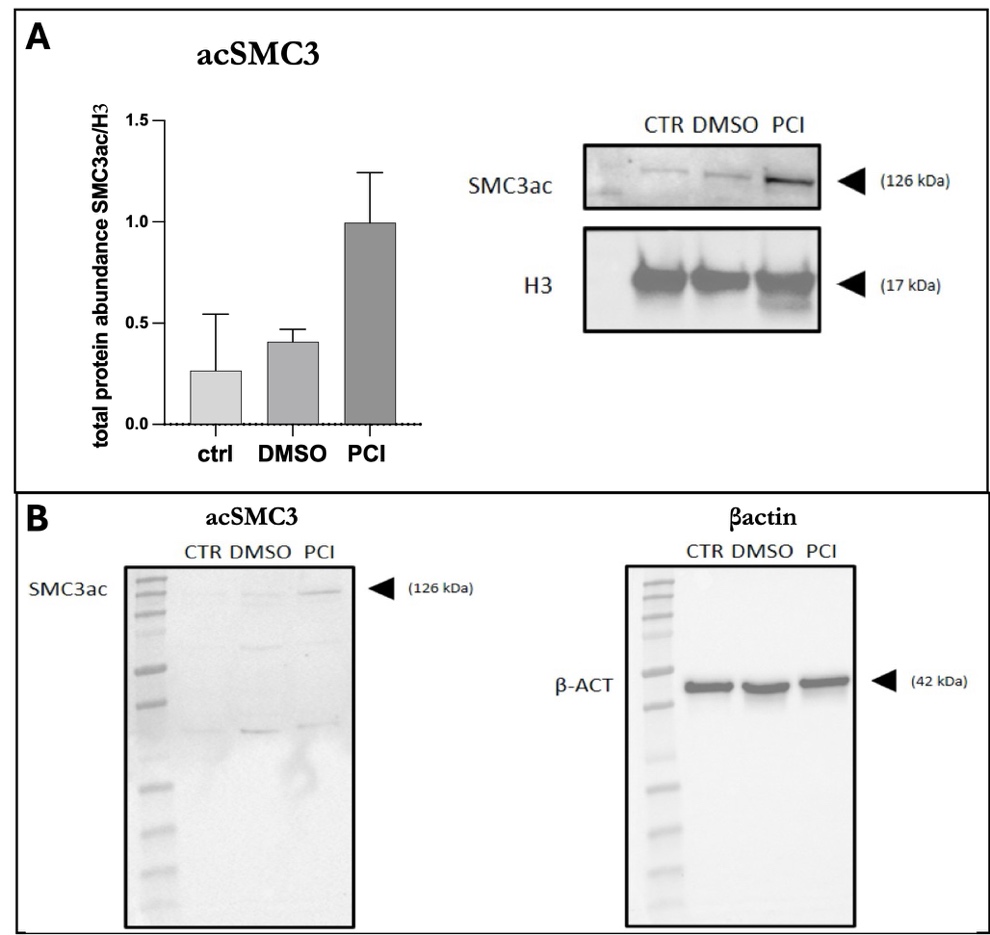


*Supplementary Fig. 1*. (A) western blot showing total protein abundance of acetylated SMC3, to show the effect on acetylation cohesion target, normalized on H3 duplicate analysis on hNPC treated with HDAC8 inhibitor PCI34051, vehicle (DMSO), and control not treated (CTRL, light grey); (B) representative western blot showing total protein abundance of acetylated SMC3 and βactin, whole membrane is shown.


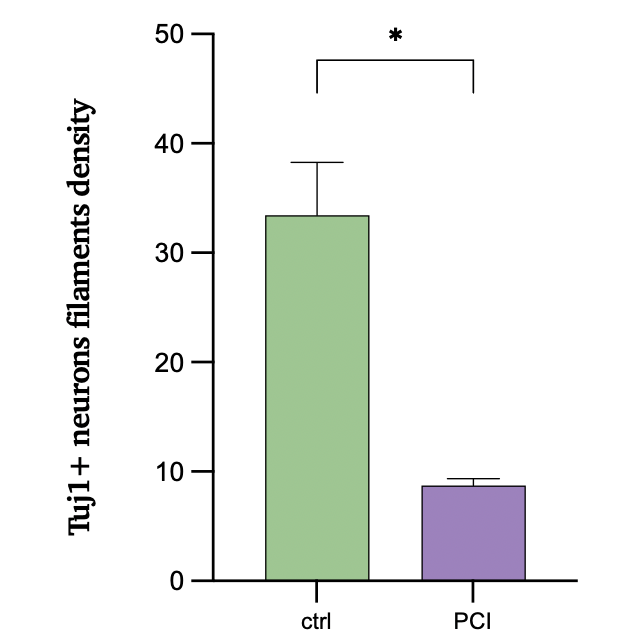


*Supplementary Fig. 2*. Immunofluorescence TUJ1 assay was used to evaluate the cytotoxicity of HDC8 inhibition in projections of hNPC CdLS models during differentiation compared to controls. Upon HDAC8 inhibition (PCI) exposure, hNPCs CdLS models show a significant decrease in TUJ1-positive filaments density. Student t test: PCI vs. ctrl p-value * 0,0342 (3 images/ condition in triplicate, counted blinded).

*References*

[1] Prinetti A, Chigorno V, Prioni S, Loberto N, Marano N, Tettamanti G, et al. Changes in the Lipid Turnover, Composition, and Organization, as Sphingolipid-enriched Membrane Domains, in Rat Cerebellar Granule Cells Developing in Vitro. Journal of Biological Chemistry 2001;276:21136–45. https://doi.org/10.1074/JBC.M010666200.

[2] Prinetti A, Basso L, Appierto V, Villani MG, Valsecchi M, Loberto N, et al. Altered sphingolipid metabolism in N-(4-hydroxyphenyl)-retinamide-resistant A2780 human ovarian carcinoma cells. J Biol Chem 2003;278:5574–83. https://doi.org/10.1074/JBC.M207269200.

[3] Scandroglio F, Loberto N, Valsecchi M, Chigorno V, Prinetti A, Sonnino S. Thin layer chromatography of gangliosides. Glycoconj J 2009;26:961–73. https://doi.org/10.1007/S10719-008-9145-5.

[4] Di Fede E, Taci E, Castiglioni S, Rebellato S, Ancona S, Grazioli P, et al. p300 inhibition delays premature cellular senescence. Npj Aging 2025;11. https://doi.org/10.1038/S41514-025-00251-Y,.

[5] Dobin A, Davis CA, Schlesinger F, Drenkow J, Zaleski C, Jha S, et al. STAR: ultrafast universal RNA-seq aligner. Bioinformatics 2013;29:15–21. https://doi.org/10.1093/BIOINFORMATICS/BTS635.

[6] Liao Y, Smyth GK, Shi W. FeatureCounts: An efficient general purpose program for assigning sequence reads to genomic features. Bioinformatics 2014;30:923–30. https://doi.org/10.1093/BIOINFORMATICS/BTT656,.

[7] Love MI, Huber W, Anders S. Moderated estimation of fold change and dispersion for RNA-seq data with DESeq2. Genome Biol 2014;15:1–21. https://doi.org/10.1186/S13059-014-0550-8/FIGURES/9.

[8] Grazioli P, Parodi C, Mariani M, Bottai D, Di Fede E, Zulueta A, et al. Lithium as a possible therapeutic strategy for Cornelia de Lange syndrome. Cell Death Discov 2021;7:1–11. https://doi.org/10.1038/S41420-021-00414-2.
