# Supplementary figures and images for: Lithium ameliorates neural differentiation restoring cell death balance in Cornelia de Lange syndrome 2D and 3D models
Source: Cell Death Discov. 2026 Mar 28;12:203. doi: 10.1038/s41420-026-03085-z (PMC13150035; doi:10.1038/s41420-026-03085-z)

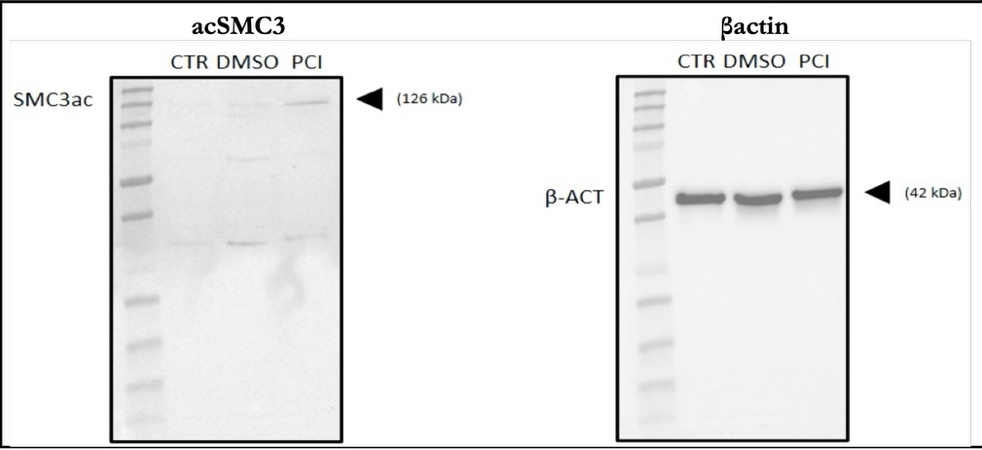

Supplement: Supplementary file 2 — whole membrane WB [file 41420_2026_3085_MOESM2_ESM.jpg]
